# Efficacy of fractional CO_2_ laser in combination with stromal vascular fraction (SVF) compared with fractional CO_2_ laser alone in the treatment of burn scars: a randomized controlled clinical trial

**DOI:** 10.1186/s13287-023-03480-8

**Published:** 2023-09-23

**Authors:** Masoumeh Roohaninasab, Fariba Khodadad, Afsaneh Sadeghzadeh-Bazargan, Najmolsadat Atefi, Sona Zare, Alireza Jafarzadeh, Seyyedeh Tahereh Rahimi, Maryam Nouri, Mohammad Ali Nilforoushzadeh, Elham Behrangi, Azadeh Goodarzi

**Affiliations:** 1 Department of Dermatology, Rasool Akram Medical Complex Clinical Research Development Center (RCRDC), School of Medicine, Iran University of Medical Sciences (IUMS), Tehran, Iran; 2 Skin and Stem Cell Research Center, Tehran University of Medical Sciences, Tehran, Iran; 3 Laser Application in Medical Sciences Research Center, Shahid Beheshti University of Medical Sciences, Tehran, Iran; 4 Stem Cell and Regenerative Medicine Institute, Sharif University of Technology, Tehran, Iran; 5 Department of Mechanical Engineering, Sharif University of Technology, Tehran, Iran; 6 Skin Repair Research Center, Jordan Dermatology and Hair Transplantation Center, Tehran, Iran

**Keywords:** Burn scar, Stromal vascular fraction, CO_2_ fractional laser, Laser, Ablative laser, CO_2_ laser, Fractional, SVF, Scar, Trial, Efficacy, Safety, Satisfaction

## Abstract

**Background:**

The appearance of skin scars is known as one of the main side effects of skin burns. Stromal vascular fraction (SVF), as a rich source of cell populations with tissue regeneration properties, plays an important role in the healing of skin lesions. Fractional CO_2_ lasers have occupied a special place in treating skin lesions, particularly skin scars, since their introduction. Our study aimed to compare the combination of SVF and fractional CO_2_ laser with fractional CO_2_ laser alone in the treatment of burn scars.

**Method:**

This double-blind clinical trial study was conducted on ten patients with burn scars that were treated three times with a fractional CO_2_ laser at site of burn lesions, and one of the two areas studied was randomly injected with SVF. Two months after completion of the procedure, patients' scars were assessed using the Vancouver scar scale (VSS), biometric criteria, and physician and patient satisfaction ratings.

**Results:**

The results confirmed a significant improvement in VSS, cutometry, R7 criteria, complete density sonography, and skin density sonography in the fractional CO_2_ laser-treated group. The VSS criteria, epidermal thickness sonography, complete density sonography, and skin density sonography in the group treated with the combination of fractional CO_2_ laser and SVF also showed significant improvement. The VSS criteria and melanin index of Mexameter in the group treated with SVF in combination with fractional CO_2_ laser were significantly better than the group treated with fractional CO_2_ laser alone. Also, physician and patient satisfaction in the group treated with SVF injection in combination with fractional CO_2_ laser was significantly higher than the other group.

**Conclusion:**

The results confirm the efficacy of SVF injection in combination with fractional CO_2_ laser in the treatment of burn scars and can be considered as a treatment option for better management of these lesions.

*Trial registration*: The study protocol was retrospectively registered at Iranian Registry of Clinical Trials with code: IRCT20210515051307N1, Registration date: 2021-11-14, URL: https://www.irct.ir/trial/56337.

## Introduction

The most common causes of skin scars are burns, wounds, striae distensae, and acne. Burns include heat contact, radiation, and chemical or electrical contact that cause damage to the skin or other tissues. Scars caused by burn damage are not only deformed, but also cause shrinkage, hypertrophic changes, and keloid tissue, which can negatively affect quality of life. These scars often result in symptoms such as itching, persistent hyperemia, chronic folliculitis, and limited range of motion [1–4].

Common treatments for burn scars include silicone gel, pressure dressings, hydration, corticosteroid injections, massage therapy, cryotherapy, and surgery. However, new and advanced methods have been developed to treat burn scars. Lasers, especially fractional and ablative non-fractional lasers, can play an important role in the treatment of scars, especially burn scars [5–7]. On the other hand, autologous fat grafting is considered a new and promising method for skin rejuvenation and scar treatment [8].

Adipose tissue is a very complex tissue containing mature fat cells, adipocyte progenitor cells, fibroblasts, pericytes, vascular smooth muscle cells, endothelial cells, monocytes, macrophages, and lymphocytes, i.e., a heterogeneous cell population called stromal vascular fraction (SVF) [9]. The SVF is obtained after processing adipose tissue. Adipose-derived stem cells (ADSC) are isolated and cultured from this heterogeneous cell population, which have the ability to differentiate into mesodermal, ectodermal, and endodermal cells. ADSC stimulate angiogenesis and reduce fibrosis by releasing growth factors, cytokines, proteins, and exosomes, resulting in improved wound healing and scar regeneration [10].

To date, there is no gold standard for the treatment of scar tissue, and treatments are mostly based on the individual experience of physicians with variable success [11].

Moreover, in the treatment of scars and keloid tissue, the combination of several therapeutic interventions has been shown to be more effective than monotherapy [12]. Therefore, given the importance of burn scars and their consequences for patients, combined CO_2_ fractional laser treatment with SVF is designed as a new treatment option in this study.

## Materials and methods

### Patients

This study was a double-blind, randomized clinical trial of patients referred to the dermatology clinic from 2021 to 2022. It included ten patients with skin types 1 to 4 who had burn scars at more than one site that had been present for at least three months and were between 25 and 50 years of age. Exclusion criteria included laser treatment in the past three months, pregnancy and breastfeeding, coagulation and platelet disorders, use of anticoagulant medications, diabetes and connective tissue disease, active viral infections, history of malignancy, and use of chemotherapeutic agents. Before the start of the study, all patients were informed about the procedure and completed the informed consent form. Their information such as age, sex, location of the scar, time of its formation, and treatments received were recorded in the questionnaire. All ten participating patients had at least two burn scars on more than one extremity. The type of burn scar was an atrophic burn scar, and its extent varied among each patient. After the initial assessment, the burn scars of all patients was randomly divided into two parts, with one part considered as the intervention area and the other as the control area. The injection area had dimensions of 5 cm in length and width.

### Randomization and blinding

The simple randomization method was used for randomization. In this way, two scar areas were randomly named A and B in each patient, and patients were given four sealed envelopes with the letters AS, AL, BS, and BL. The first letter represents the desired area, and the second letter indicates that the procedure can be performed. If the letter S is present, the SVF injection is performed along with the fractional CO_2_ laser, and if the letter L is present, only the fractional CO_2_ laser is injected along with normal saline as a placebo. Once the procedure is established, the other area will undergo a different procedure than the specified area. This study is a double-blind clinical trial where the patient and physician evaluate the results, and the statisticians do not know which treatment was used for which lesion. Normal saline was injected as a placebo to blind the patients.

### Preparation of SVF

First, 100 cc of fat was removed from the thigh area of each patient. The tissue was then washed with phosphate-buffered saline (PBS) (Miltenyi Biotec, Cologne, Germany) to remove red blood cells and leukocytes. The adipose tissue was digested with collagenase type I (Worthington Biochemical Corp, Lakewood, USA) for 20 min at 37 °C to produce a collagenase solution with a concentration of 0.1%. Enzyme digestion was prevented by washing with DMEM 10% FBS (Invitrogen, Carlsbad, USA), and floating and lysed fat cells were discarded. SVF cells were pelleted by centrifugation at 500*g* for 10 min. The pellet was resuspended in PBS, and an erythrocyte lysis buffer (Sigma-Aldrich Corp, St. Louis, USA) was added and incubated at 37 °C for 10 min. This cell suspension was centrifuged (500*g*, 5 min), and SVF cells were counted using an automatic cell counter.

The viability of isolated SVF cells was evaluated in the laboratory using an automatic cell counter. Flow cytometry was performed to analyze the surface marker expression of SVF cells. The data analyses were conducted using Partec—CyFlow ML. Data analysis was carried out using FloMax® software.

### CO_2_ fractional laser settings

Patients were treated in the burn areas with a fractional CO_2_ laser (SmartXide DOT®, DEKA, USA), choosing a power 13, a stack 2, a spacing 800 µm, and a scanning dwell time 900 microseconds.

### Intervention methods

In all patients, CO_2_ laser alone (together with injection of normal saline as placebo) was performed in one part of the burn scar, and the combination of CO_2_ laser and SVF injection was performed in the other lesion. The duration of treatment is three sessions, one month apart, as follows: First session: fractional CO_2_ laser for both lesions with placebo injection in one lesion and SVF in the other lesion, second session: fractional CO_2_ laser only for both lesions, and the third session: fractional CO_2_ laser for both lesions accompanied by placebo injection in one lesion and SVF injection in the other.

### Assessment method

**Fig. 1:**
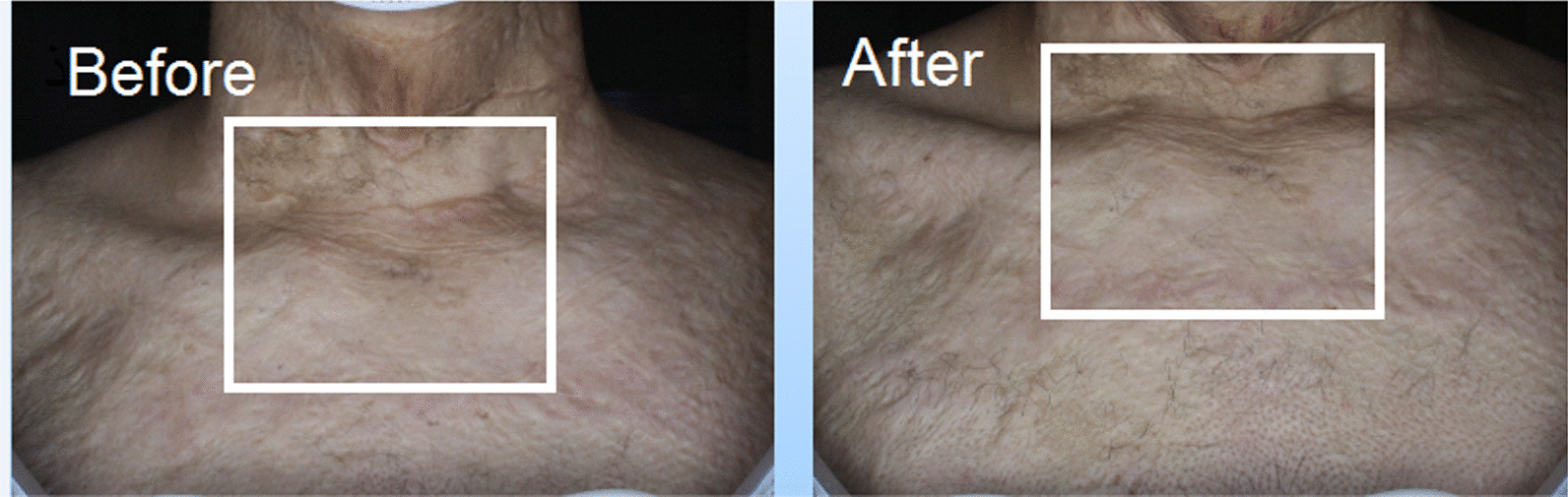
Skin burn scar before and after treatment with fractional CO_2_ laser
  The sum of all scores is examined to evaluate the improvement of the lesion (Table 1).

All patients were examined before the start of the study and two months after completion of the study as described below:

1. Determination of the Vancouver scar scale for both groups, which assesses four characteristics of the lesion, including vascularity, pigmentation, height, and flexibility. According to the items listed in Fig. 1, a score is given for each characteristic.

**Table 1:** Vancouver scar scale [13]

	Scar characteristic	Score
Vascularity	Normal	0
	Pink	1
	Red	2
Purple	Normal	3
	Pigmentation	0
	Hypopigmentation	1
	Hyperpigmentation	2
Pliability	Normal	0
	Supple (flexible with minimal resistance)	1
	Yielding (giving way to pressure)	2
	Firm (inflexible, not easily moved)	3
	Banding (rope like blanches with extension of scar, does not limit range of motion)	4
	Contracture (permanent shortening of scar producing deformity, limits range of motion)	5
Height	Flat	0
	$\ll$ 2 mm	l
	$\gg$ 2–5 mm	2
	5 mm	3
Total score		13
2. Biometric assessment in both groups before and 2 months after completion of treatment. For this purpose, the following quantitative parameters and a probe with a frequency of 75 Hz were used:
  Corneometer: to measure tissue hydration (stratum corneum layer).
  Mexameter: to measure the amount of melanin and erythema of the lesion.
  Tewameter: to measure transepidermal water loss.
  Colorimeter: to measure color changes of the skin.
  Cutometer: to determine the elasticity of the tissue and includes the following parameters:
  R2: viscoelasticity.
  R5: pure elasticity.
  R7: percentage of immediate recovery compared to amplitude after suction.
3. Patient and physician satisfaction with treatment in both groups is based on the overall assessment of patients and physicians and includes the options of No Response, Little, Somewhat, Good, and Excellent, which are rated as 0, 1, 2, 3, and 4 points, respectively.

### Data analysis

Data were analyzed using SPSS statistical software. Results for quantitative variables were expressed as mean ± SD and for qualitative variables as percentage. Normality of the distribution of variables was checked using the K–S test of the SPSS software, and depending on whether the variable of interest was quantitative or qualitative, it was examined using the Mann–Whitney *U* test, the Student t test, or the chi-square test between two groups. Numerical values with a *p* value of less than 5% were considered statistically significant. All data were analyzed using SPSS version 22 software. Regression models were used to test for association with control of confounding factors.

## Results

**Fig. 2:**
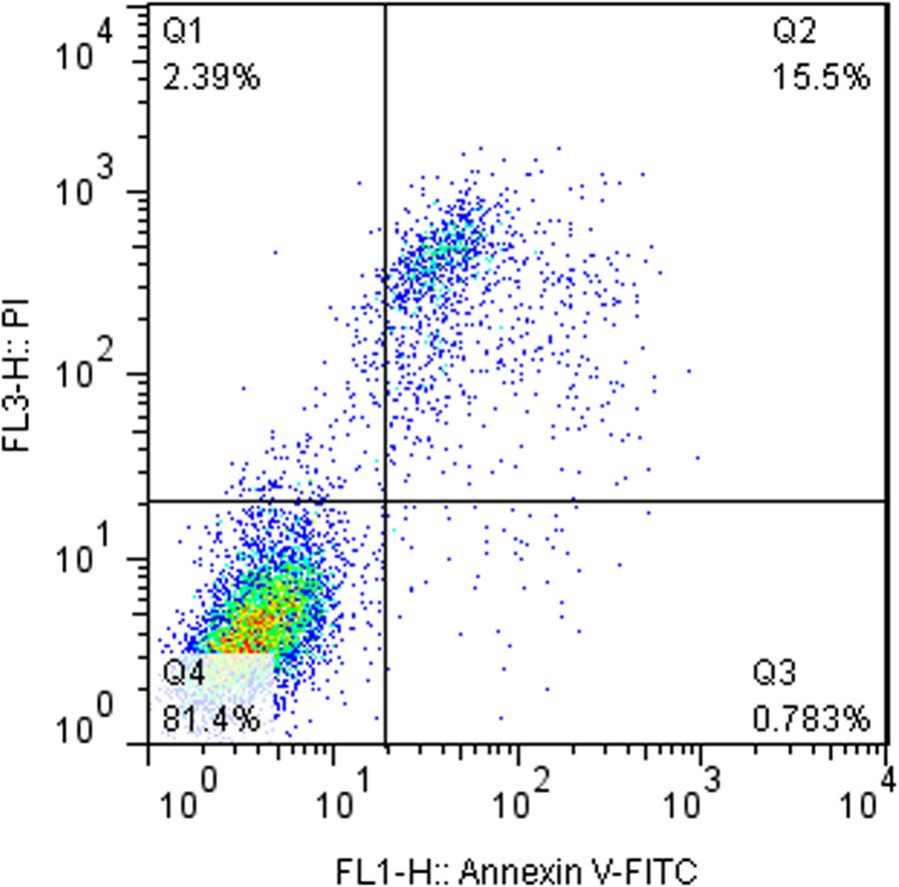
Viability of SVF cells (prepared for injection)

**Fig. 3:**
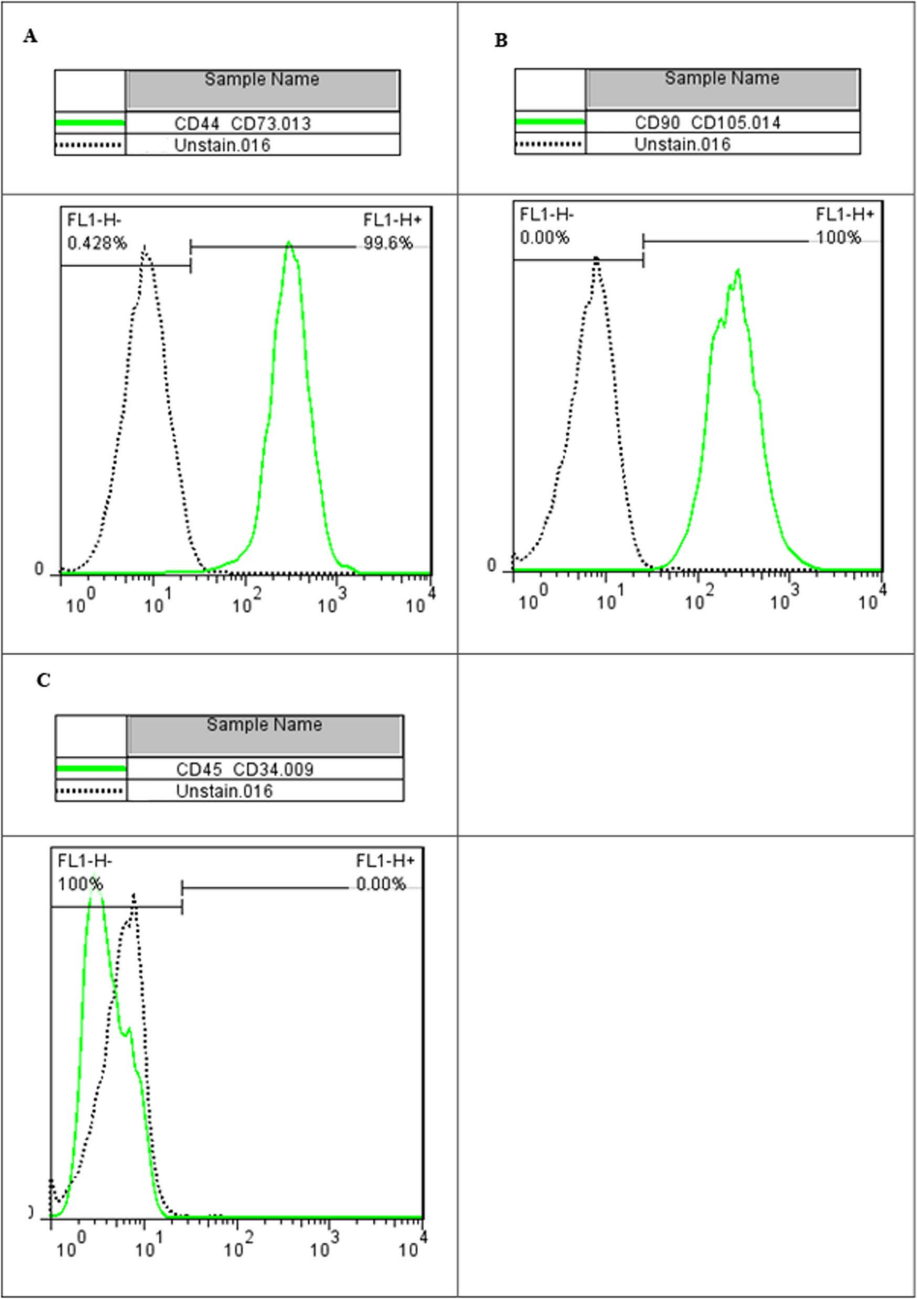
SVF cells CD44, CD73 (**A**) CD90, CD105 (**B**) surface markers and had very small expression against hematopoietic cell markers CD34/CD45 (**C**)

The mean yield of cells was 20 × 106 cells/mL from aspirated 100 cc of fat tissue, and the mean viability of these cells was 81.4%. SVF cell surface markers were evaluated using flow cytometry. The results demonstrated that the isolated SVF cells expressed CD44, CD90, CD105, and CD73 surface markers, while showing minimal expression of hematopoietic cell markers CD34/CD45 (Figs. 2, 3).

**Fig. 4:**
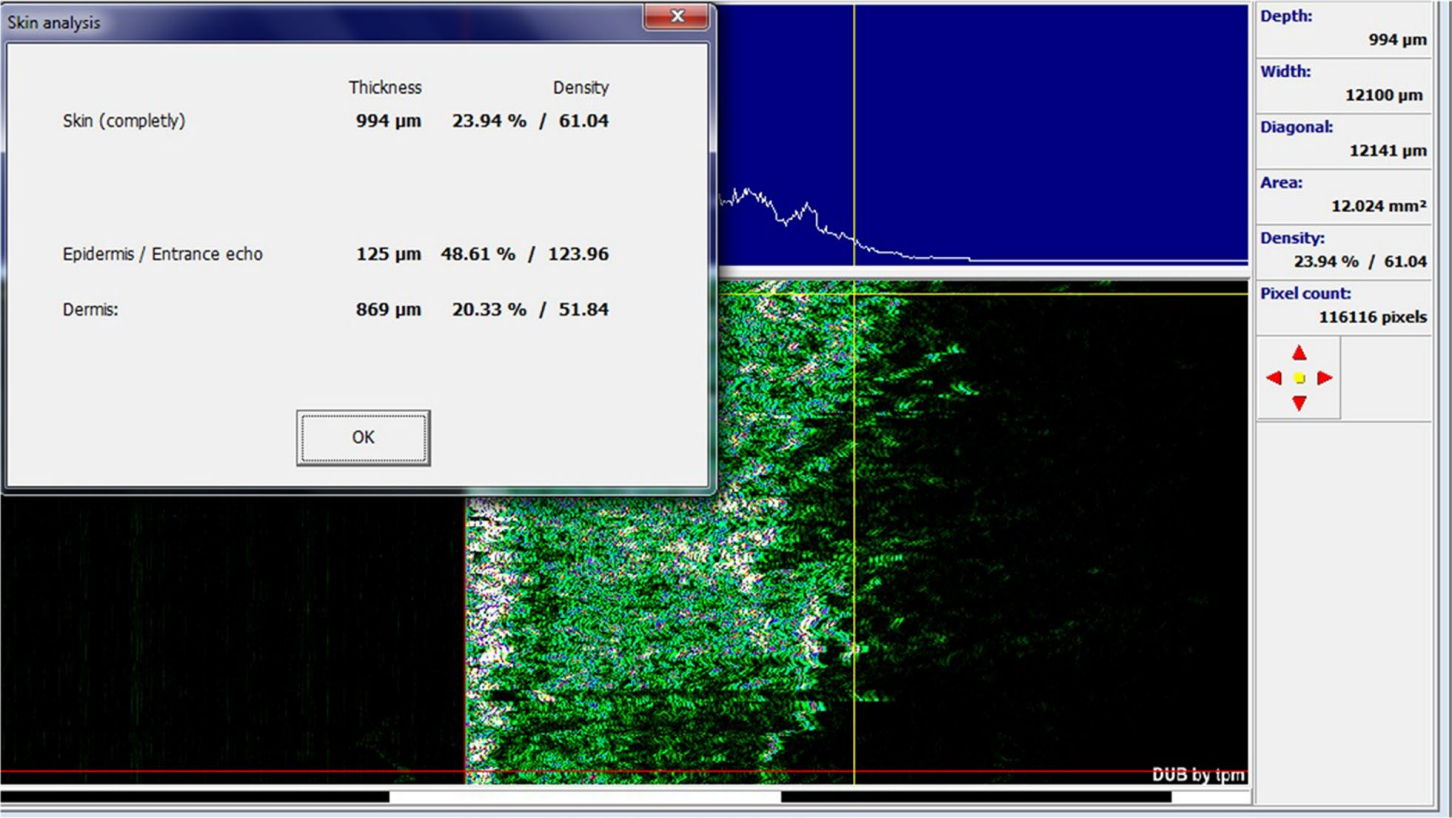
Sonographic findings before intervention with fractional CO_2_ laser

**Fig. 5:**
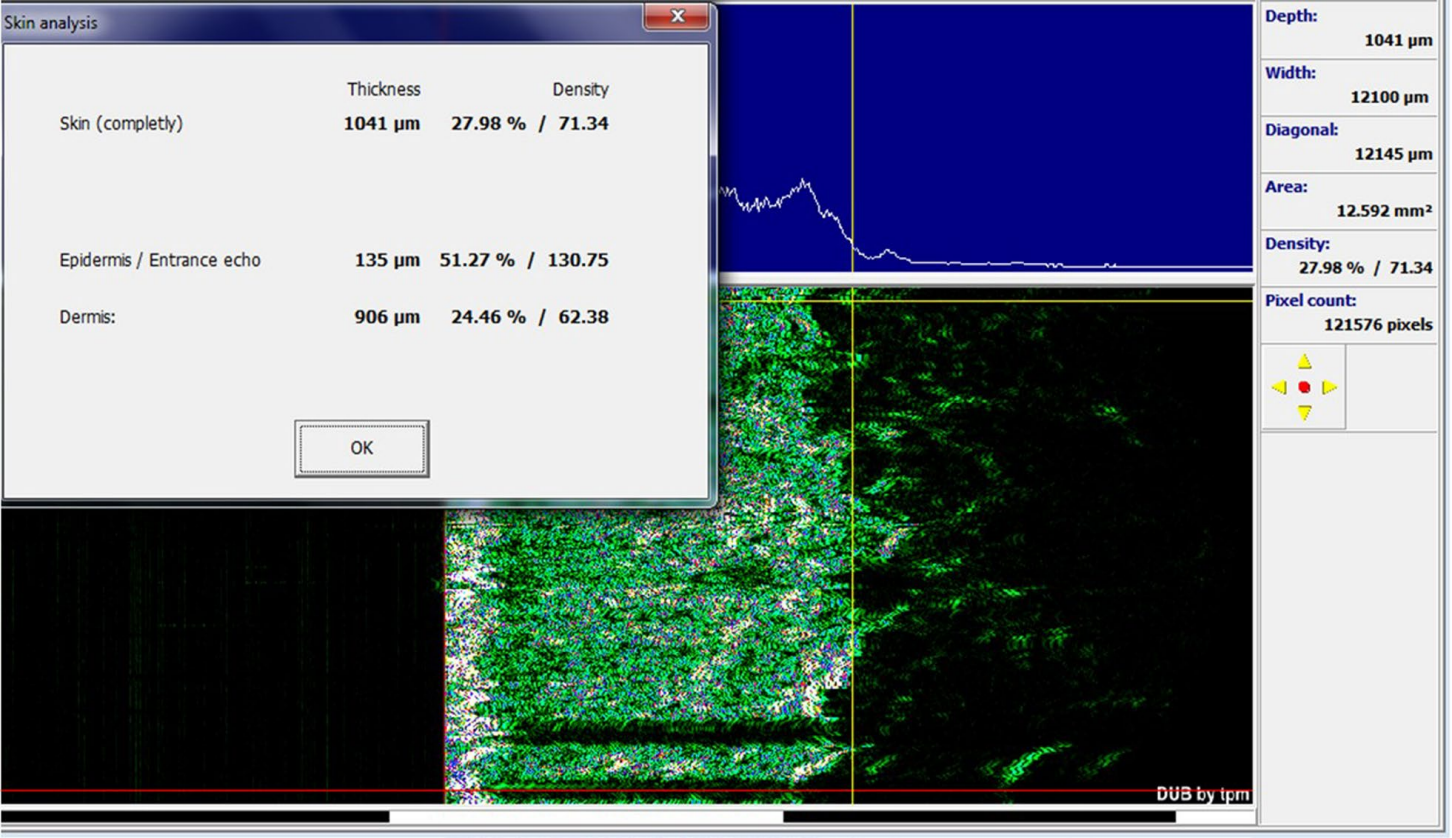
Sonographic findings after intervention with fractional CO_2_ laser

The average age of the studied subjects was 31.00 ± 9.67 years. Among the studied subjects, seven subjects (70.0%) were women and the rest were men. In the group treated with fractional CO_2_ laser, there was a significant improvement between the two groups before and after the procedure in the mean variables of the Vancouver scar scale (7.40 ± 1.35 vs. 5.90 ± 1.97, *p* value = 0.007), cutometry R7 (0.59 ± 0.17 vs. 0.48 ± 0.13, *p* value = 0.032), complete density sonography (10.89 ± 6.04 vs. 17.27 ± 8.19, *p* value = 0.018), and dermal density sonography (7.95 ± 5.29 vs. 14.18 ± 8.46, *p* value = 0.020) (Figs. 1, 4, 5).

**Fig. 6:**
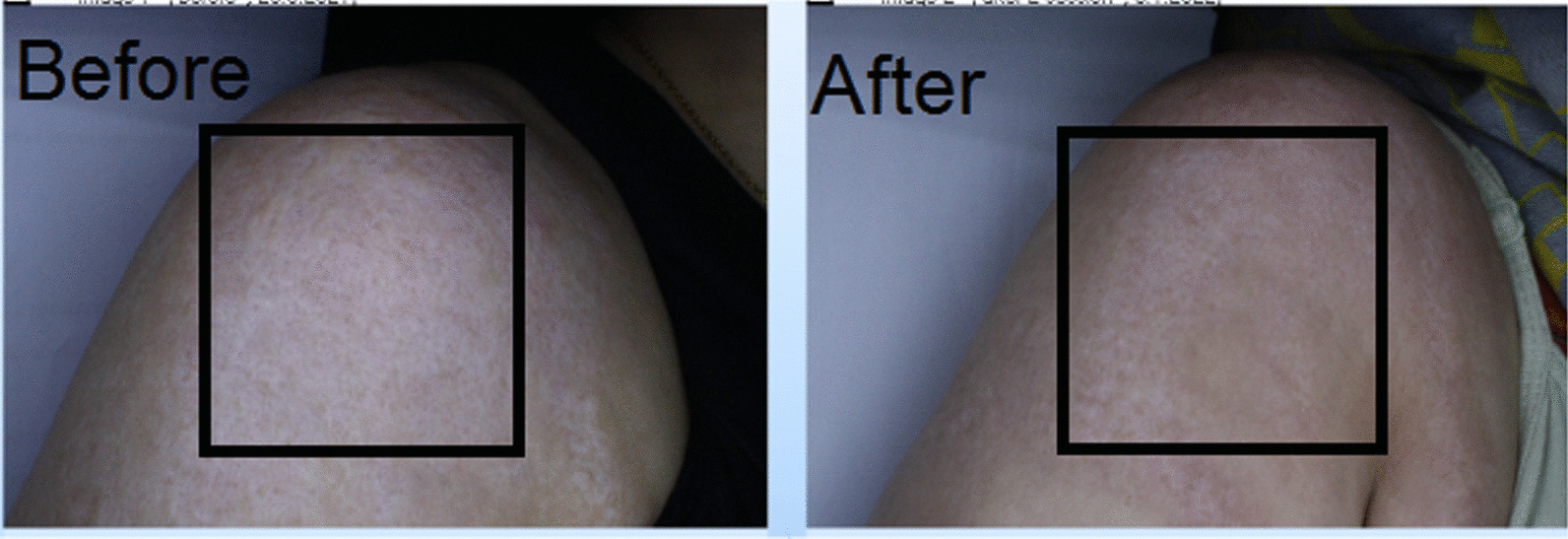
Skin burn scar before and after treatment with SVF and fractional CO_2_ laser

**Fig. 7:**
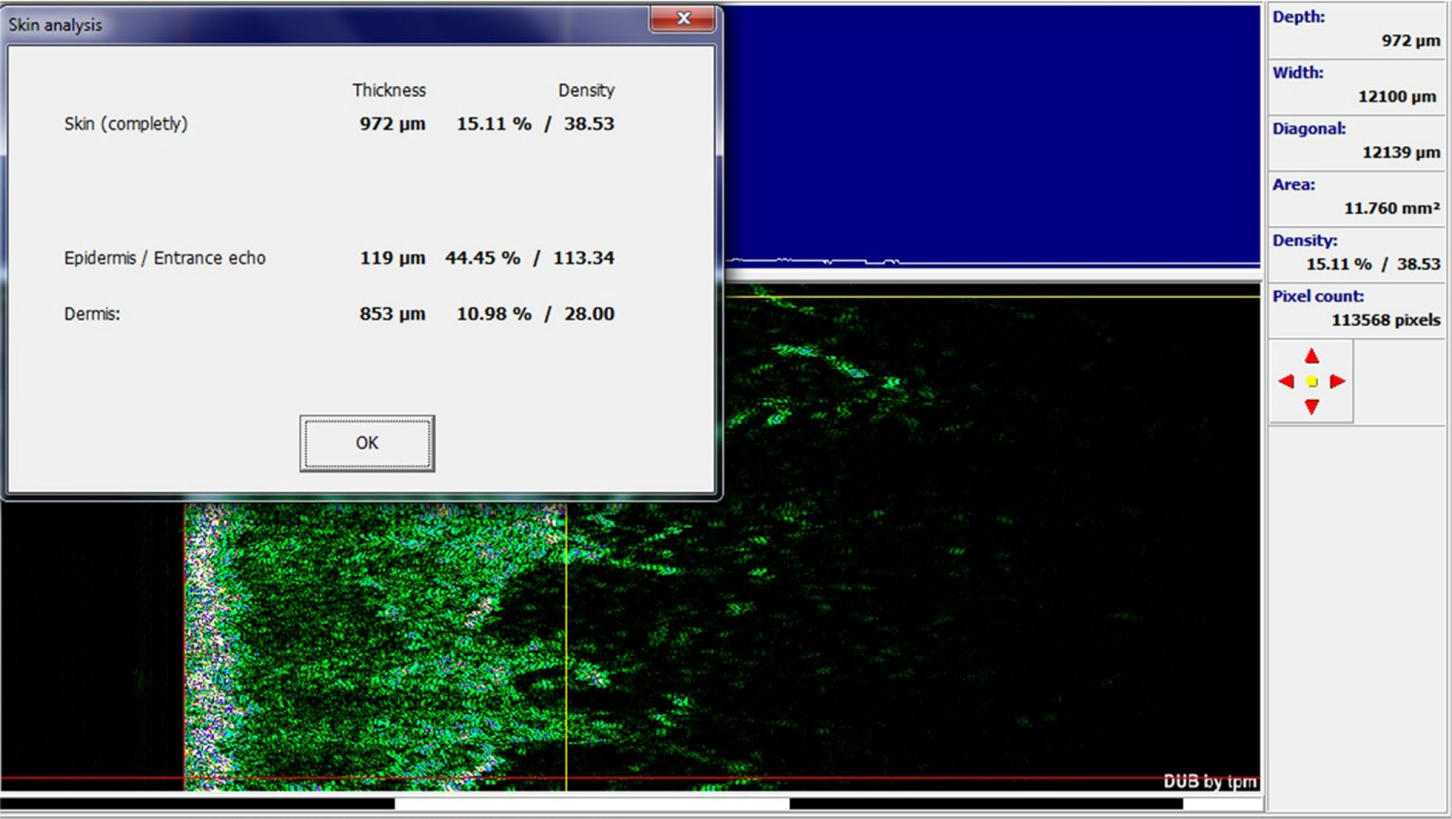
Sonographic findings before intervention with fractional CO_2_ laser and SVF

**Fig. 8:**
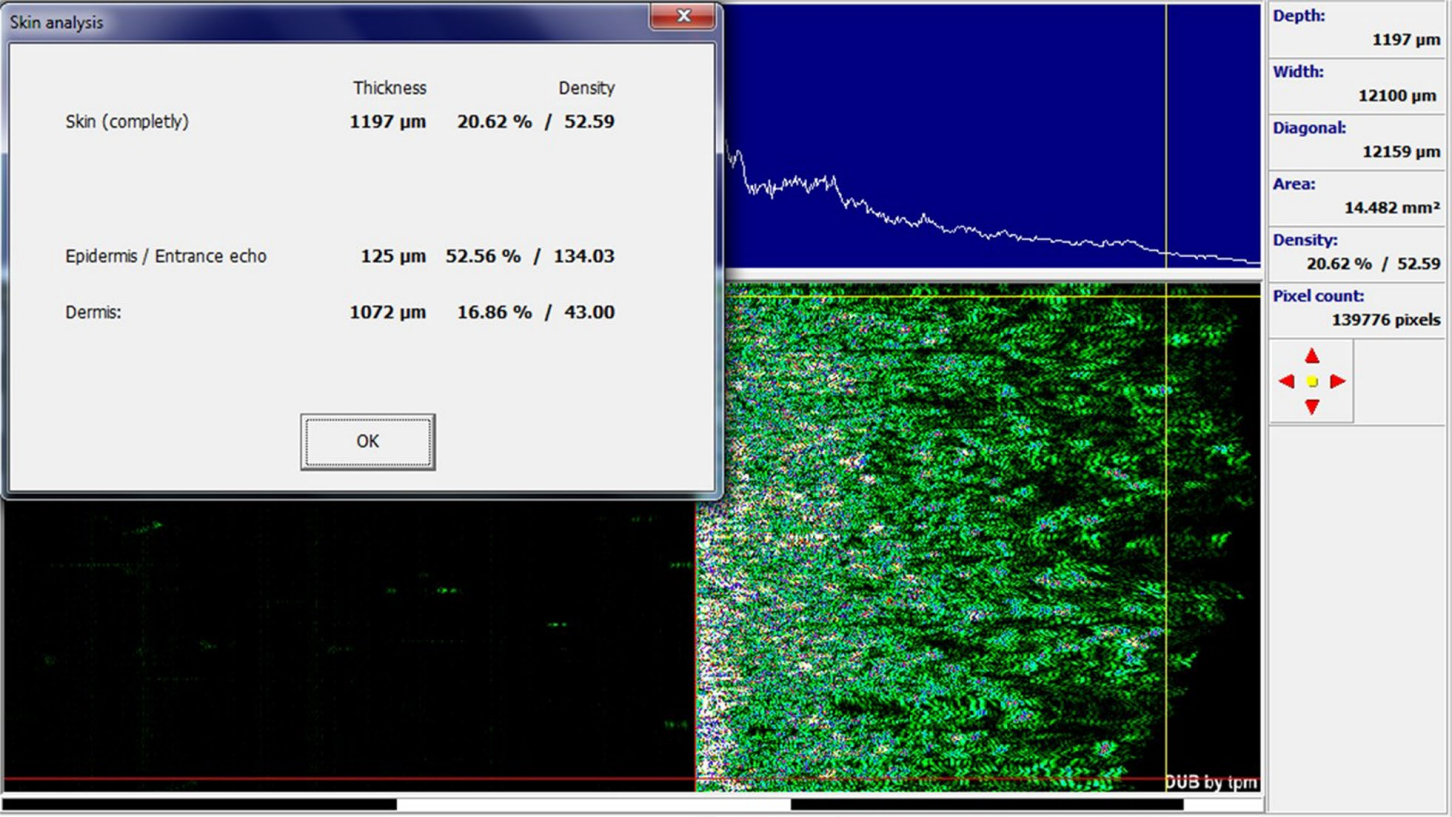
Sonographic findings after intervention with fractional CO_2_ laser and SVF

In the group treated with CO_2_ laser in combination with SVF, there was a significant improvement between the two groups before and after the procedure in the mean Vancouver scar scale variables (7.50 ± 1.35 vs. 4.80 ± 1.03, *p* value < 0.0001), epidermal thickness sonography (99.00 ± 15.50 vs. 111.50 ± 14.83, *p* value  = 0.016), complete density sonography (12.01 ± 8.71 vs. 21.50 ± 5.97, *p* value  = 0.003), and skin density sonography (9.32 ± 8.23 vs. 17.92 ± 6.40, *p* value  = 0.002) (Figs. 6, 7, 8).

**Fig. 9:**
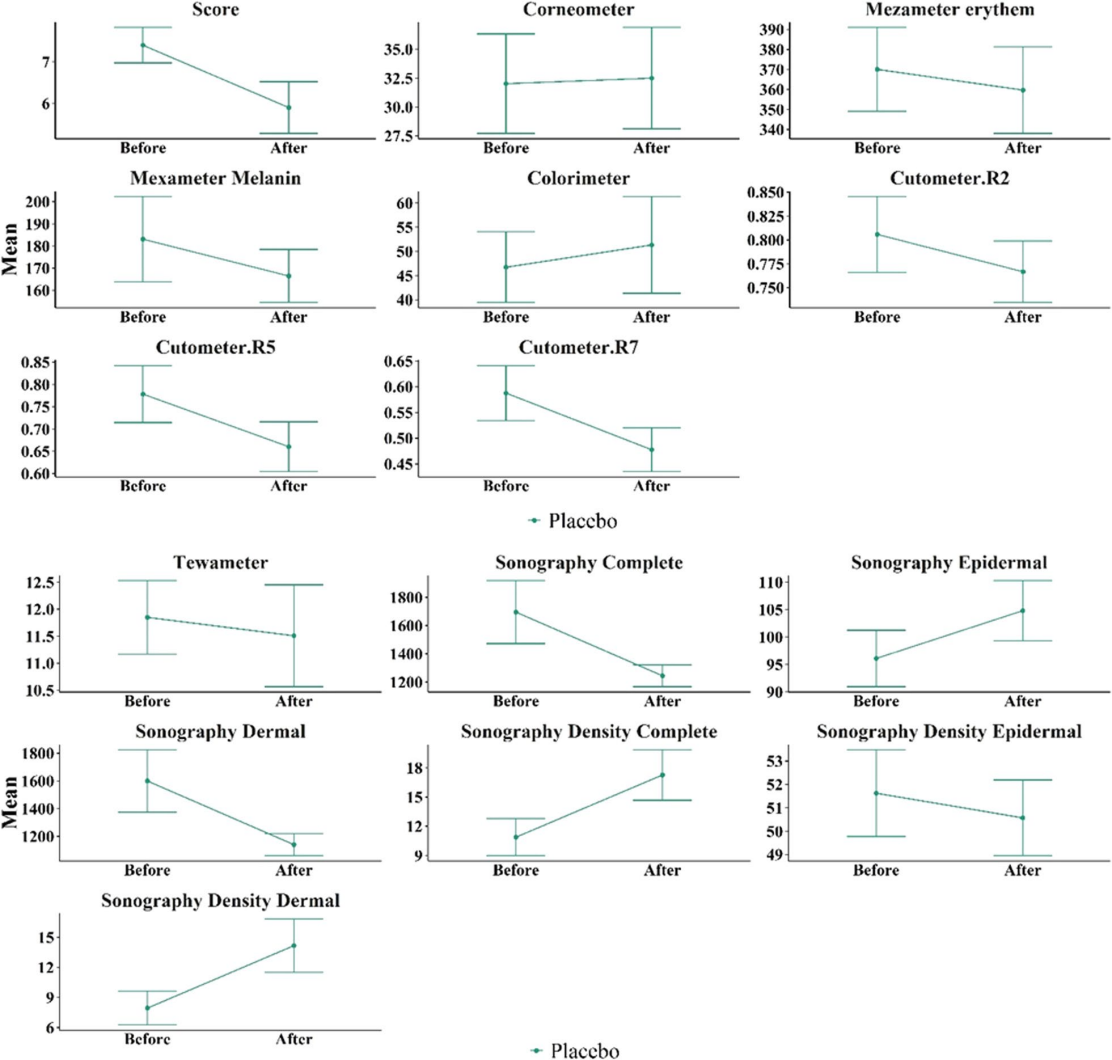
Comparison of the average amount of biometric variables of burn scar severity in the group treated with fractional CO_2_ laser

**Fig. 10:**
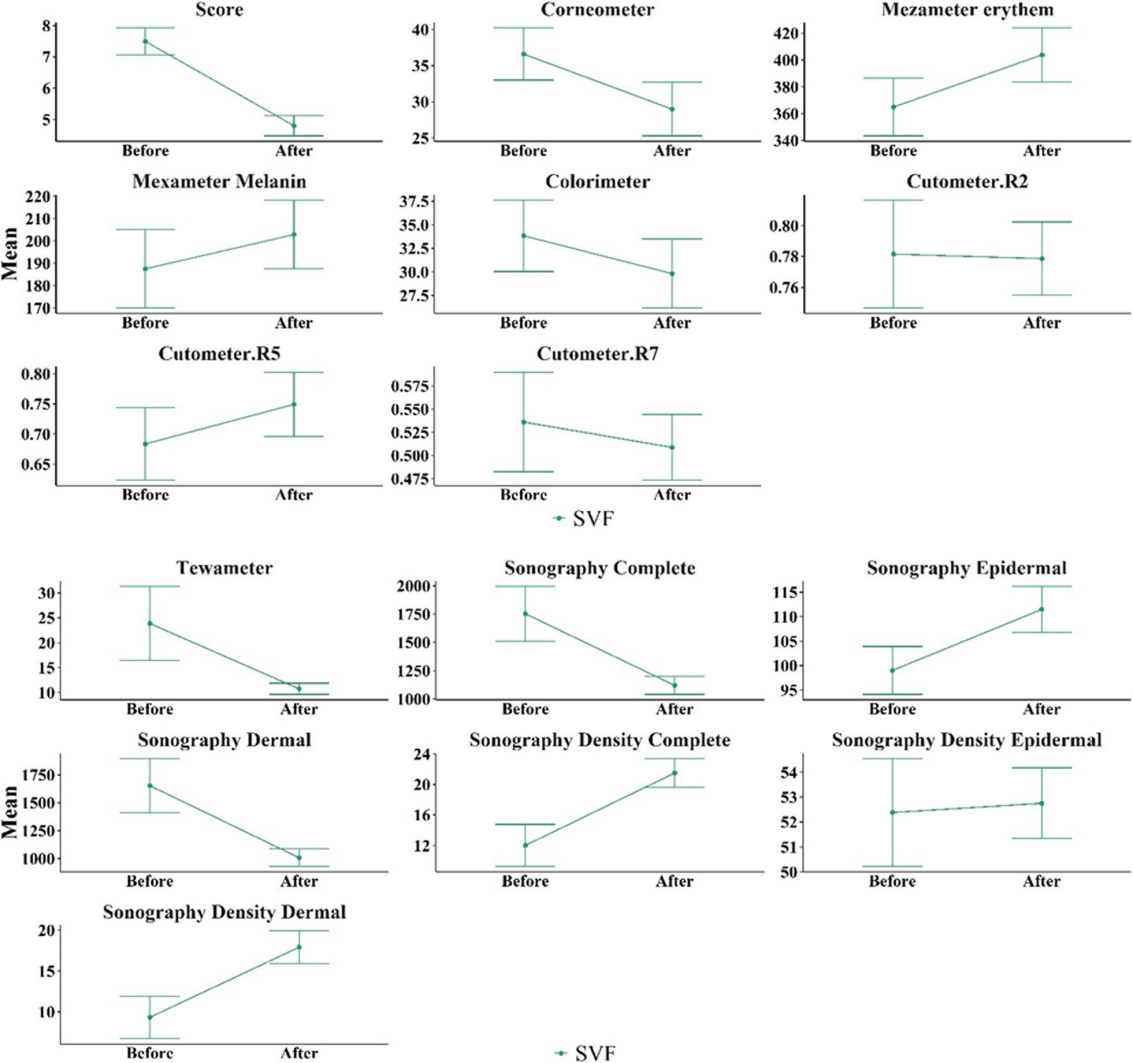
Comparison of the average amount of biometric variables of burn scar severity in the group treated with fractional CO_2_ laser combined with SVF injection

We will now compare in more detail the variables related to burn scar severity between patients treated with fractional CO_2_ laser and patients treated with SVF injection in combination with fractional CO_2_ laser. For this comparison, we first considered the primary variables for burn scar severity before the procedure as baseline values (Tables 2, 3, Figs. 9, 10). Figure 9 displays the average variables in the intervention group treated with CO_2_ fractional laser before and after the intervention. The error bars represent the mean ± standard deviation. Similarly, Fig. 10 presents the average variables in the intervention group treated with SVF alongside CO_2_ fractional laser before and after the intervention. Again, the error bars represent the mean ± standard deviation.

**Table 2:** Comparison of the average amount of burn scar severity variables before and after the intervention in the group treated with CO_2_ fractional laser

Variable	Before intervention	After intervention	Difference of after and before value	*p* value (paired sample *t* test)	*p* value (Wilcoxon signed rank test)
Vancouver scar scale	7.40 ± 1.35	5.90 ± 1.97	1.50 ± 1.35	0.007	0.004
Tewametry index	11.85 ± 2.15	11.51 ± 2.99	0.34 ± 3.73	0.780	0.846
Corneometry index	32.02 ± 13.61	32.50 ± 13.86	− 0.48 ± 13.43	0.913	0.770
Erythema index of Mexameter	370.03 ± 66.41	359.67 ± 68.52	10.37 ± 5576	0.571	0.945
Melanin index of Mexameter	183.11 ± 60.85	166.47 ± 37.77	16.64 ± 37.93	0.199	0.432
Colorimetry	46.73 ± 23.06	51.34 ± 31.64	− 4.61 ± 38.24	0.780	0.313
Cutometry R2	0.81 ± 0.13	0.77 ± 0.10	0.04 ± 0.12	0.347	0.375
Cutometry R5	0.78 ± 0.20	0.66 ± 0.18	0.12 ± 0.17	0.057	0.084
Cutometry R7	0.59 ± 0.17	0.48 ± 0.13	0.11 ± 0.14	0.032	0.027
Complete thickness sonography	1695.80 ± 704.96	1244.60 ± 244.53	451.20 ± 632.38	0.05	0.232
Epidermal thickness sonography	96.10 ± 16.28	104.80 ± 17.31	− 8.70 ± 13.92	0.08	0.051
Dermal thickness sonography	1599.70 ± 713.87	1139.80 ± 253.71	459.90 ± 635.82	0.048	0.232
Complete density sonography	10.89 ± 6.04	17.27 ± 8.19	− 6.38 ± 7	0.018	0.004
Epidermal density sonography	51.63 ± 5.87	50.57 ± 5.12	10.06 ± 4.90	0.511	0.695
Dermal density sonography	7.95 ± 5.29	14.18 ± 8.46	− 6.24 ± 6.96	0.02	0.006

**Table 3:** Comparison of the average amount of burn scar severity variables before and after the intervention in the group treated with CO_2_ fractional laser in combination with SVF injection

Variable	Before intervention	After intervention	Difference of after and before value	*p* value (paired sample *t* test)	*p* value (Wilcoxon signed rank test)
Vancouver scar scale	7.50 ± 1.35	4.80 ± 1.03	2.70 ± 0.82	< 0.001	0.002
Tewametry index	23.88 ± 23.53	10.74 ± 3.56	13.14 ± 22.82	0.102	0.029
Corneometry index	36.62 ± 11.43	28.99 ± 11.74	7.63 ± 13.43	0.101	0.160
erythema index of Mexameter	364.97 ± 67.99	403.83 ± 63.44	− 38.87 ± 64.20	0.088	0.084
melanin index of Mexameter	187.57 ± 55.67	202.90 ± 48.75	− 15.33 ± 28.11	0.199	0.064
colorimetry	33.83 ± 11.99	29.83 ± 11.59	4 ± 7.73	0.261	0.313
Cutometry R2	0.78 ± 0.11	0.78 ± 0.07	0.00 ± 0.1	0.931	0.922
Cutometry R5	0.68 ± 0.19	0.75 ± 0.17	− 0.07 ± 0.19	0.314	0.432
Cutometry R7	0.54 ± 0.17	0.51 ± 0.11	0.03 ± 0.18	0.648	0.625
Complete thickness sonography	1752.40 ± 764.51	1120.40 ± 252.62	632 ± 632.38	0.061	0.193
Epidermal thickness sonography	99.00 ± 15.50	111.50 ± 14.83	− 12.50 ± 13.29	0.016	0.020
Dermal thickness sonography	1653.40 ± 769.54	1008.90 ± 250.49	644.50 ± 929.55	0.056	0.160
Complete density sonography	12.01 ± 8.71	21.50 ± 5.97	− 9.49 ± 7.53	0.003	0.004
Epidermal density sonography	52.38 ± 6.86	52.76 ± 4.49	− 0.37 ± 5.85	0.846	0.770
Dermal density sonography	9.32 ± 8.23	17.92 ± 6.40	− 8.61 ± 6.39	0.002	0.004

**Fig. 11:**
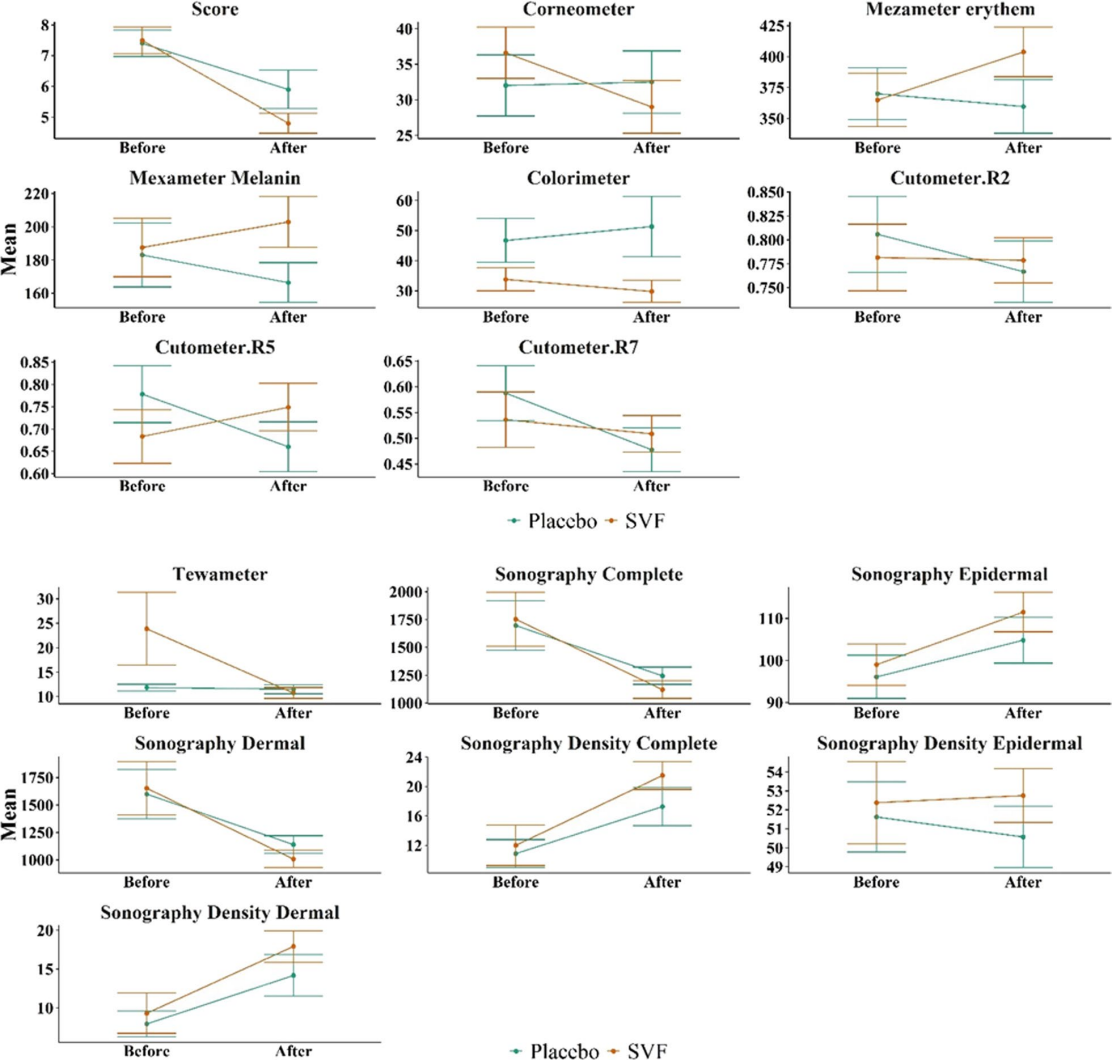
Comparison of the average amount of biometric variables of burn scar severity in the group treated with fractional CO_2_ laser combined with SVF injection and the group treated with fractional CO_2_ laser alone

Comparing the two aforementioned groups, Vancouver scar scale and Mexameter melanin index were significantly better in the group treated with SVF and fractional CO_2_ laser than in the group treated with fractional CO_2_ laser only. To this end, the difference between the Vancouver scar scale and Mexameter's melanin index was significant when controlling for the values of before. When controlling the value of Vancouver scar scale before treatment, the mean Vancouver score in SVF group is 1.81 units lower than placebo group (*p* value = 0.032). When controlling for Mexameter melanin index, the average Mexameter melanin index is 33.69 units higher in the SVF group than in the control group (*p* value = 0.009) (Fig. 11).

In the group treated with a fractional CO_2_ laser in combination with SVF, patient and physician assessment scores were higher than in the group treated with a fractional CO_2_ laser alone, and this difference was significant (Table 4).

**Table 4:** Comparison of the average amount of patient and physician global assessment score between group treated with SVF in combination with CO_2_ fractional laser and group treated with CO_2_ fractional laser alone

variable	group	Mean ± standard deviation	Median	*p* value (independent *t* test)	*p* value (exact Mann–Whitney *U* test)
Patient global assessment score	Treated with SVF in combination with CO_2_ fractional laser	3.20 ± 0.92	3.5	0.009	0.019
Patient global assessment score	Treated with CO_2_ fractional laser	2.10 ± 0.74	2	0.009	0.019
Physician global assessment score	Treated with SVF in combination with CO_2_ fractional laser	3.40 ± 0.52	3	< 0.001	< 0.001
Physician global assessment score	Treated with SVF in combination with CO_2_ fractional laser	2.20 ± 0.42	2	< 0.001	< 0.001

## Discussion

From the introduction of SVF to the present day, numerous therapeutic applications have been reported, including its effective role in the treatment of retinopathy and nerve regeneration [14, 15], as well as its effective role in the treatment of osteochondral disease and the treatment of myocardial infarction [16, 17]. On the other hand, the use of fractional CO_2_ lasers is widely applied in dermatology, such as in the treatment of striae distensae [18–21], atrophic acne scars [22–25], and burn scars [26–28].

Several studies have been performed on the methods used to treat skin scars [1, 5, 8, 9]. Among the methods used to treat skin scars in previous studies, the role of fractional CO_2_ laser should be mentioned [22, 23, 25, 28, 29]. Previous studies have also mentioned the efficacy of SVF in the treatment of skin scars [30–32]. Our study investigated the efficacy of combining the above two methods compared with using fractional CO_2_ laser alone for burn scars. The results of our study, which was designed as a double-blind clinical trial, showed significant improvement in burn scars in the group treated with fractional CO_2_ laser based on the average Vancouver scar scale, cutometer R7, complete density sonography, and skin density sonography. On the other hand, in the group treated with fractional CO_2_ laser along with SVF injection, the improvement of burn scar was significant based on average Vancouver scar scale, epidermal thickness sonography, complete density sonography, and dermal density sonography. And when comparing the above two groups based on the variables of Mexameter melanin index, Vancouver scar scale, and overall patient and physician assessment, the group treated with SVF injection combined with fractional CO_2_ laser showed a significant difference in burn scar healing.

Comparing the results of the present study with similar studies, a study of facial scars caused by trauma or burns conducted by Gentil et al. in Italy in 2014 showed that 63% of scars had healed in the group treated with SVF after one year, compared with 39% in the control group and 69% in patients treated with platelet-rich plasma (PRP) and nanofat [33].

In our study, the additional injection of SVF to fractional CO_2_ laser treatment was associated with an improvement in burn scar severity variables, which is consistent with the results of the above study on the role of SVF in the treatment of skin scars, including burn scars.

Other studies have been conducted on the performance of fractional CO_2_ laser in the treatment of skin scars. In one of these studies, the results of a meta-analysis showed that the fractional CO_2_ laser significantly improved the Vancouver scar scale score (VSS). Patient and physician scar rating scales also showed significant improvements with fractional CO_2_ laser treatment. In addition, the fractional CO_2_ laser significantly decreased the thickness of the scar measured by ultrasound [29]. In our study, the group treated with the fractional CO_2_ laser showed significant improvement in the Vancouver scar scale, patient global assessment score, and physician global assessment score.

In other results of this study, only R2 index (scar elasticity) in cutometry improved significantly with laser treatment, but measurement of R0 index (scar firmness) showed no significant improvement [29]. In our study, ultrasound and biometric findings are presented in more detail, and complete density sonography, dermal density sonography, and cutometry R7 were evaluated, which confirmed a significant difference after laser treatment.

In our study, the efficacy of SVF was also evaluated. There is an evaluation that was not investigated in the above study. In our study, there was no significant difference in the R2 variable in cutometry in patients before and after laser. This difference might be due to the different nature of the study. In contrast to the aforementioned study, our study was a clinical study, whereas the aforementioned study was a systematic review.

In a 2019 study conducted in South Korea, Kim and colleagues examined a skin defect on a patient's leg that occurred after an accident. The aforementioned lesion was covered by a skin graft, but the resulting scar was prominent and developed into a hypertrophic scar. In this study, the patient was treated with fractional CO_2_ laser in five sessions, and one month later, he was treated with SVF injection and ablative CO_2_ laser simultaneously. The result of the examination after one year of follow-up showed that the surface of the scar had been flattened and the pigment deposits had been removed [34]. The results of this study were also consistent with our study on the simultaneous use of fractional CO_2_ laser and SVF injection in scar healing.

In two separate studies, Lee and his colleagues in South Korea investigated the effect of SVF injection in the surgical treatment of depressed scars. In the first study, 17 patients underwent SVF injection concurrently with surgical scar reduction. In the second study, seven patients underwent scar revision surgery concurrently with SVF injection, and eight patients underwent surgical treatment alone as a control group. The OSAS (Observer Scar Assessment System), SBSES (Stony Brook Evaluation System), VSS (Vancouver Scar Scale), and VAS (Visual Analog Scale) scoring systems were used to evaluate response to treatment. All patients showed significant improvement based on all 4 scoring systems. Patients in the SVF group had a higher cure rate than the non-SVF group on all scoring systems except SBSES. The highest rate of improvement was also seen in scar height and flexibility, whereas a significant change was seen in vascularity [35].

In addition to the above studies, some studies have also investigated the effect of fractional CO_2_ laser in the treatment of other types of skin scars [22, 23, 25]. Nilforoushzadeh and colleagues compared two treatment methods, including fractional CO_2_ laser alone and fractional CO_2_ laser combined with subcision, in the treatment of atrophic acne scars in a clinical trial. In this study, patients with atrophic acne scars were treated with two methods, including fractional CO_2_ laser alone (5 sessions 3 weeks apart) on the right side of the face and fractional CO_2_ laser combined with subcision (subcision combined with laser in the first session and 4 fractional CO_2_ laser sessions 3 weeks apart after 3 weeks) on the left side of the face. Patient satisfaction was measured 6 months after treatment, and side effects were compared in two treatment groups. The results showed that therapeutic efficacy was 54.7% for the combined method and 43% for the fractional CO_2_ laser alone method. Mean patient satisfaction based on the method VAS (visual analog scale) was 6.6 for the combined method and 5.2 for the laser alone method. Erythema formation was observed with both methods. PIP (Postinflammatory pigmentation) and hyperpigmentation were observed only with the combined method, but after 6 months, complications did not occur in either group. The results show that the combined method achieved more effective results in improving scars and patient satisfaction. The researchers concluded that this method can be used as an efficient treatment method. However, complications such as bruising and hyperpigmentation occurred with this method, which did not occur with laser treatment alone [36]. The above results are in agreement with our study regarding the efficacy of CO_2_ fractional laser in the treatment of skin scars. On the other hand, it has been pointed out that the simultaneous application of multiple treatment methods is more effective in healing scars. In our study, the results confirmed the more effective use of SVF injection in combination with fractional CO_2_ laser compared to fractional CO_2_ laser alone in the treatment of burn scars.

Another study by Khan Ali and his colleagues showed that fractional CO_2_ laser was more effective than microneedling in reducing patients' acne scar scores. In addition, few side effects occurred with either treatment method. In addition, there was no significant difference in physician and patient satisfaction with either method [37]. The results of this study are consistent with our study regarding the efficacy of CO_2_ fractional laser in the treatment of skin scars. Therefore, it can be concluded that CO_2_ fractional laser is effective in the treatment of both burn scars and acne.

In Galal and colleagues' study of 30 patients with acne scars, the two methods of fractional CO_2_ laser alone and fractional CO_2_ laser combined with platelet-rich plasma injection (PRP) were compared, and the results of the study showed significant improvement in scar depth on both sides of the patients' faces. However, the simultaneous use of laser and PRP resulted in greater improvement in patients than laser alone. And although 70% of the patients in this study had a dark skin type, no hyperpigmentation was noted in the patients [38]. The results of this study are also consistent with our study in terms of both the efficacy of using fractional CO_2_ laser in scar treatment and the greater efficacy of the combined treatment compared to laser treatment alone, and in our study, the combined treatment of SVF injection and fractional CO_2_ laser was a significant difference in improvement in terms of Vancouver scar scale, Mexameter melanin index, patient global assessment score, and physician global assessment score compared to fractional CO_2_ laser treatment alone.

In the study by Behrangi et al. performed in 2022 on seven patients with acne scar complaints, all patients were treated with SVF injection in one half of the face, while the other half of the face served as a control group. The results of the evaluation after 3 months confirmed a significant improvement in sonographic variables such as skin thickness and total skin thickness compared to baseline values at the beginning of the study. In contrast, the improvement in the sonographic variable of epidermis thickness was not significant [30]. In our study, the results in the group treated with SVF in combination with fractional CO_2_ laser confirmed a significant improvement in melanin Mexameter and Vancouver scar scale compared with fractional CO_2_ laser alone. There was no significant difference in the changes in skin thickness and total thickness of skin variables in the two groups. The existence of this difference could be due to the time interval between intervention and assessment. In the aforementioned study, the period between SVF injection and evaluation was three months, whereas in our study, this period was two months. On the other hand, part of this difference could be due to the different nature of the scars. The mentioned study was performed on acne scars, while in our study, burn scars were investigated.

Finally, it is important to mention that several studies have mentioned the efficacy of SVF in the treatment of various diseases as well as different skin lesions, including burn scars and acne scars [30, 32, 35]. The therapeutic effect of SVF is based on several mechanisms, including angiogenesis, inhibition of apoptosis, and anti-inflammatory effect [39]. And it seems that its efficacy in healing burn scars in our study is also consistent with the above effects. Based on the results of this study and other studies, the combined treatment method of CO_2_ laser and SVF seems to be more effective than other methods in treating scars caused by burns or other factors. The above method can be used as an alternative method in the treatment of these lesions. It should be mentioned that one of the limitations of the present study is the small sample size, which may affect the results. Therefore, conducting clinical trials with a larger sample may provide more reliable results.

## Conclusion

The results of the present study show that the combined treatment of fractional CO_2_ laser and SVF as a new treatment has acceptable efficacy in the treatment of burn scars. Also, the patient and physician satisfaction with the treatment of this method was higher than that of the control group. Based on the above results, it can be concluded that the application of this method can be used as an effective method in the treatment of all types of skin scars, especially burn scars. It is worth mentioning that due to the small sample size in the current study, it is recommended to conduct a similar study with a larger sample to confirm the results.

## Data Availability

The data that support the findings of this study are available from the corresponding author, [M.A.N], upon reasonable request.

## Abbreviations

SVF: Stromal vascular fraction
VSS: Vancouver scar scale
ADSC: Adipose tissue-derived stem cells
PBS: Phosphate-buffered saline
